# Clinical and echocardiographic differences in patients with dilated cardiomyopathy in the frame depending preserved left ventricular contractile reserve

**DOI:** 10.1186/1749-8090-8-S1-P159

**Published:** 2013-09-11

**Authors:** V Peric, P Otasevic

**Affiliations:** 1 University of Pristina, School of Medicine, Internal Clinic, Kosovska Mitrovica, Serbia; 2 Dedinje Cardiovascular Institute, Belgrade, Serbia

## Background

Way and the importance of the assessment of left ventricular contractile reserve in patients with dilated cardiomyopathy, as well as differences between patients with and without preserved left ventricular contractile reserve, the current problems that are being intensively studied.

## Methods

We studied 55 consecutive patients with dilated cardiomyopathy (mean age 55±10 years, 49 (89.1%) male). All patients underwent exercise stress-echocardiography on tredmil using modified Bruce protocol. Contractile reserve was assessed by measuring changes in wall motion score index (ΔWMSI) at rest and at peak exercise.

## Results

Preserved contractile reserve had 19 patients (34.5%). Patients with and without preserved left ventricular contractile reserve were no different in age, gender, disease duration, NYHA class, presence of atrial fibrillation and left bundle branch block. Patients with preserved contractile reserve had a smaller end-diastolic (62.95 ± 5.64 vs. 68.58 ± 7.53 mm, p = 0.006) and end-systolic left ventricular dimension (47.63 ± 6.06 vs. 55.39 ± 8.13 mm, p = 0.001), lower left ventricular mass index (159.68 ± 31.90 vs. 197.06 ± 54.04 g/m2, p = 0.008), a higher slope Vp measured by color M-mode (50.66 ± 17.28 vs.. 43.53 ± 18.73 cm / s, p = 0.024 ), higher systolic mitral annular displacement measured by tissue Doppler (6.02 ± 1.67 vs. 4.88 ± 1.26 cm / s, p = 0.006), smaller end-diastolic (70.94 ± 24.45 vs. 94.92 ± 33.13 ml/m2, p <0.001) and end-systolic left ventricular volume index (52.71 ± 22.52 vs. 78.71 ± 33.04 ml/m2, p <0.001), higher left ventricular ejection fraction (27.10 ± 6.83% vs. 18.67 ± 7.28%, p <0.001) and lower wall motion score index (2.19 ± 0:23 vs. 2:46 ± 0:28, P = 0.001).

## Conclusions

Patients with preserved left ventricular contractile reserve were morphologically and functionally less damaged left ventricle.

